# Praziquantel efficacy and post-treatment reinfection and incidence of *Schistosoma mansoni* among school-aged children in Northern Uganda: a 24-week cohort study in a high transmission focus

**DOI:** 10.1186/s12879-026-13757-x

**Published:** 2026-06-06

**Authors:** John Paul Byagamy, Robert Opiro, Margaret Nyafwono, Harriet Angwech, Geoffrey Maxwell Malinga, Richard Echodu, Emmanuel Igwaro Odongo-Aginya

**Affiliations:** 1 Department of Environment and Natural Resources Management, Faculty of Agriculture and Environment, Gulu University, Gulu City, Uganda; 2 Department of Biology, Faculty of Science, Gulu University, Gulu City, Uganda; 3 Department of Microbiology and Immunology, Faculty of Medicine, Gulu University, Gulu City, Uganda; 4 Department of Environmental Health and Disease Control, Faculty of Public Health, Lira University, Lira City, Uganda

**Keywords:** *Schistosoma mansoni*, Praziquantel efficacy, Reinfection, Incidence, School-aged children, Uganda, Sub-Saharan Africa

## Abstract

**Background:**

Human schistosomiasis is a parasitic disease of major public health importance in sub-Saharan Africa. Mass drug administration (MDA) with praziquantel (PZQ) is the cornerstone of schistosomiasis control. However, its effectiveness is challenged by variable cure rates and rapid reinfection. Longitudinal data on reinfection and true incidence, which are critical for refining control strategies, are scarce from high-transmission foci like the Lango sub-region in Northern Uganda. This study assessed the efficacy of PZQ and the 24-week dynamics of reinfection and incidence of *Schistosoma mansoni* among school-aged children in this endemic area.

**Methods:**

A 24-week prospective cohort study was conducted among 802 primary school children (aged 5–16 years). At baseline, participants were screened for *S. mansoni* using the modified Kato-Katz technique and allocated into an infected cohort (*n* = 279) and an uninfected cohort (*n* = 523). The infected cohort received a single 40 mg/kg PZQ dose, with efficacy (cure rate-CR, egg reduction rate-ERR) assessed at 4 weeks. Treatment failures received a second dose. To measure true incidence, the uninfected cohort was re-screened at 4 weeks to identify and exclude prepatent infections at baseline. Reinfection in cured children and incidence in consistently negative children were assessed at 24 weeks.

**Results:**

The baseline prevalence was 34.8% (95% CI: 31.5–38.3). After the first PZQ dose, the cure rate was 82.1% (95% CI: 77.0-86.4), and the egg reduction rate was 32.9%. A second dose administered to non-responders achieved a 93.9% cure rate (95% CI: 83.6–97.9). At 24 weeks, the reinfection rate among cured children was 7.7% (95% CI: 4.9–11.8). After excluding prepatent infections (5.9%, 95% CI: 4.1–8.3) detected at 4 weeks, the true incidence of new infections was 5.0% (95% CI: 3.3–7.4) over the 24-week period.

**Conclusions:**

Praziquantel remains highly effective for treating *S. mansoni* in northern Uganda, and a second dose is valuable for curing initial treatment failures. However, the considerable burden of infection at baseline, coupled with notable reinfection and incidence rates within six months, signals persistent high transmission. Operationally, annual MDA may be insufficient; we advocate for piloting more frequent treatment (e.g., biannual MDA), integrating test-and-re-treat strategies (with careful cost-effectiveness evaluation), and strengthening complementary WASH and snail control interventions.

**Supplementary Information:**

The online version contains supplementary material available at 10.1186/s12879-026-13757-x.

## Background

Schistosomiasis is a parasitic disease of profound public health importance, classified among the most debilitating Neglected Tropical Diseases (NTDs) [1]. It is caused by trematode worms of the genus *Schistosoma*, with *Schistosoma mansoni* being the primary cause of intestinal schistosomiasis [2]. The global burden is staggering; recent estimates from the Global Burden of Disease Study 2019 indicate that schistosomiasis causes over 1.4 million disability-adjusted life years (DALYs) annually, predominantly in sub-Saharan Africa [3]. The World Health Organization (WHO) reports that over 251 million people required preventive chemotherapy in 2021, with school-aged children bearing the highest burden of morbidity [4]. In Uganda, schistosomiasis is hyperendemic, with *S. mansoni* widespread along the lake shores and river basins, including the Lake Albert and Lake Kyoga systems, which have long been documented as significant transmission foci [5–7]. A national mapping exercise confirmed endemicity in 91 of 135 districts, affecting millions of people and placing a significant strain on the country’s healthcare system [5, 6].

The pathology of *S. mansoni* infection arises from the immune response to entrapped eggs in the host tissues, leading to granulomatous inflammation and fibrosis [2]. In children, chronic infection is particularly detrimental, causing a range of morbidities including abdominal pain, diarrhea, blood in stool, and hepatosplenomegaly [8, 9]. Perhaps most critically, chronic schistosomiasis is associated with cognitive impairment, anaemia, and growth stunting, which collectively undermine educational attainment and future economic productivity [10]. This makes school-aged children not only the most infected group but also the one that suffers the most significant long-term consequences, perpetuating a cycle of poverty and disease [11].

The cornerstone of global schistosomiasis control is preventive chemotherapy through Mass Drug Administration (MDA) programs [4]. Praziquantel (PZQ), the only widely available drug for treatment, is highly effective, safe, and low-cost, making it the drug of choice for MDA [12]. The WHO recommends a single oral dose of 40 mg/kg PZQ for school-aged children in endemic areas [4]. The primary goals of MDA are to reduce the prevalence of heavy-intensity infections and associated morbidity, and to interrupt transmission over the long term [13]. The success of this strategy has been documented in several settings, leading to significant reductions in prevalence and intensity of infection [14, 15].

Despite its successes, the reliance on single-dose PZQ MDA faces several persistent challenges. First, reports of variable and sometimes suboptimal cure rates (CR) have emerged from different geographical areas [16–19]. While CRs are often high, factors such as pre-treatment infection intensity, initial worm burden, and possibly nascent drug tolerance can influence individual treatment outcomes [19–23]. This has led to discussions about the potential need for a second (repeat) dose of PZQ for individuals who fail to cure after initial treatment [24].

Second, and perhaps more fundamentally, is the challenge of rapid reinfection. The benefits of MDA can be quickly eroded in environments where transmission remains intense due to persistent contamination of water bodies with infected human excreta and frequent human-water contact [4, 25–28]. Reinfection rates can be substantial, sometimes returning to pre-treatment levels within 6–12 months, indicating that annual MDA may be insufficient in high-transmission foci [29, 30]. Understanding the rate and determinants of reinfection is therefore critical for evaluating and tailoring intervention strategies.

Third, to accurately gauge the force of transmission in a community, it is essential to measure the incidence of new infections (the rate at which previously uninfected individuals acquire the infection). A rigorous cohort study that treats and excludes prepatent cases is necessary to obtain a true measure of incidence [4, 31, 32].

The Lango sub-region in Northern Uganda is a known endemic focus for *S. mansoni* [33–35]. The population’s reliance on fishing, agriculture, and domestic activities involving open water sources like lakes Kyoga and Kwania creates perennial exposure risks [27, 34, 35]. Previous studies in the region have documented high prevalence rates among school-aged children, confirming intense transmission [34]. However, most previous studies are cross-sectional and therefore cannot distinguish between reinfection and new infection. Furthermore, a single baseline survey may miss prepatent infections (recent infections in which worms have not yet matured into egg-laying adults), leading to underestimation of the true baseline infection rate and subsequent overestimation of incidence if these individuals are misclassified [4, 31, 32].

The use of a rigorous diagnostic method, such as the modified Kato-Katz technique employed in this study [36], within a cohort design that treats and excludes prepatent cases, is therefore necessary to obtain a true measure of incidence. Filling this knowledge gap is imperative for refining schistosomiasis control in Northern Uganda and similar settings. Data on reinfection and incidence will reveal the speed at which the parasite population rebounds post-treatment, informing the optimal frequency of MDA. Evaluating the efficacy of a second PZQ dose can guide clinical management and policy regarding non-responders.

In the present study, only a small proportion of children (< 5%) reported having received PZQ in the three months preceding the baseline survey, despite residing in an area targeted by the national control program. Therefore, this 24-week cohort study was conducted to provide a comprehensive assessment of the post-treatment dynamics of *S. mansoni* in high-risk primary school children.

## Methods

### Study area and population

**Fig. 1:**
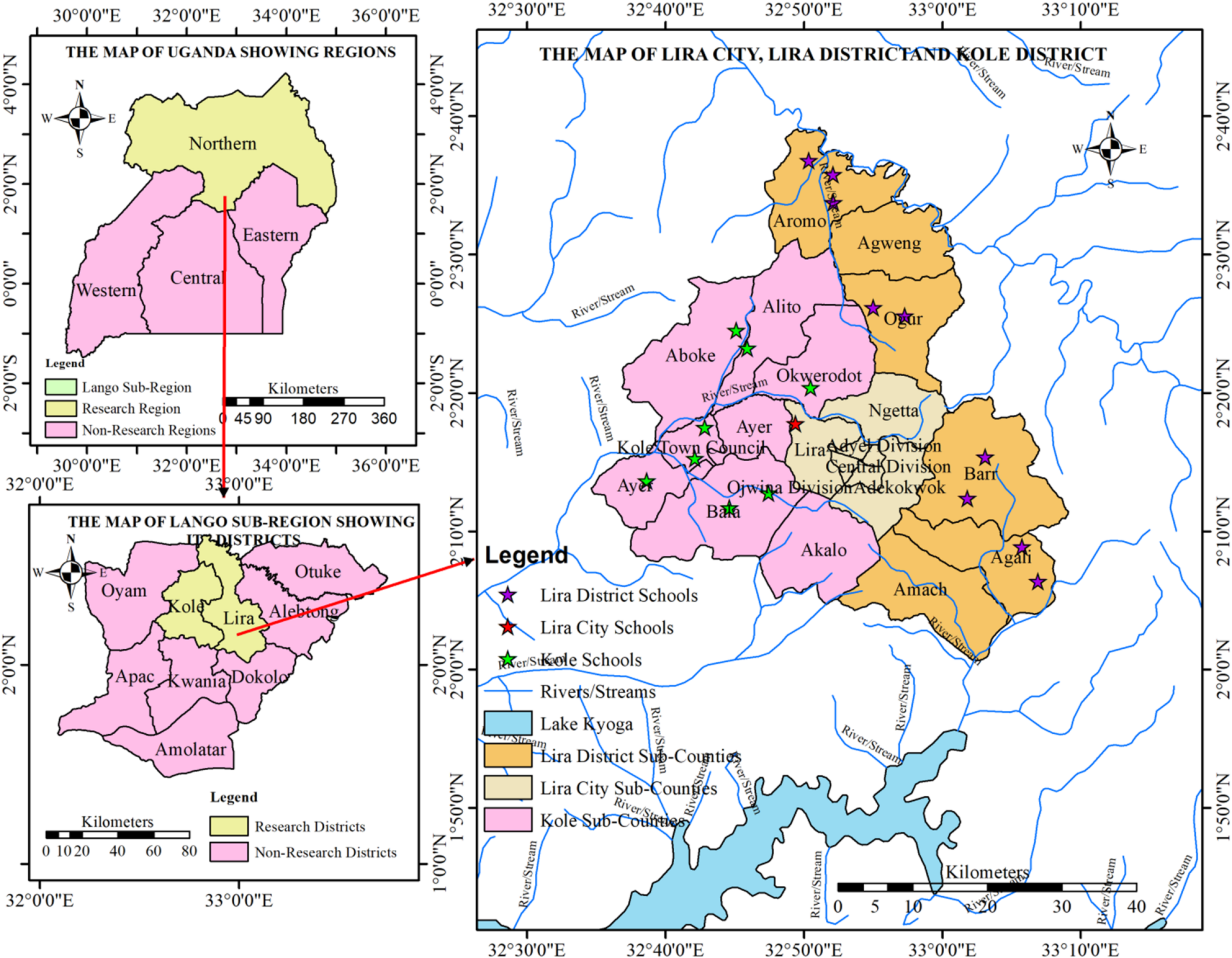
Map showing the study area and schools sampled. Administrative boundary shapefiles were obtained from DIVA-GIS (https://diva-gis.org/data.html) and are based on GADM administrative boundaries; shapefile license information is available at https://diva-gis.org/index.html. School point locations represent sampled primary schools in Lira District, Lira City, and Kole District. Purple, red, and green star symbols denote sampled schools in Lira District, Lira City, and Kole District, respectively. Rivers/streams and Lake Kyoga are shown for geographic context

This 24-week cohort study was conducted in the Lango sub-region, located in Northern Uganda. The study specifically targeted three administrative units: Lira District, the newly created Lira City, and Kole District (Fig. 1) [37]. These areas are situated in a zone known for the endemic transmission of *S. mansoni*, largely due to the presence of multiple water bodies, including Lake Kyoga and its surrounding wetlands, which serve as potential habitats for the intermediate snail host [33, 35].

Lake Kyoga, which borders the eastern edge of Lira District and Lira City, is a known focus of *S. mansoni* transmission due to the presence of *Biomphalaria* snail intermediate hosts along its shorelines and in adjacent wetlands [33]. The lake’s influence on transmission likely explains the higher prevalence observed in Lira District (44.3%) compared to Kole District (27.9%), which is further from the lake’s primary shoreline. Lira City, despite its proximity to the lake, had the lowest prevalence (18.6%), possibly due to better sanitation infrastructure and reduced human-water contact compared to rural areas.

The Lango sub-region is predominantly inhabited by the Lango ethnic group. According to the most recent national census, the combined population of the three study areas is approximately 726,675 people [37]. The primary economic activities are subsistence and commercial agriculture (cultivating crops like sesame (*Sesamum indicum*), groundnuts, and maize), small-scale fishing, and local trade [38]. The climate is tropical, characterized by two distinct rainy seasons (March-May and August-October), which influence water body levels and potentially modulate transmission dynamics and human-water contact patterns [39].

The study population consisted of primary school children aged 5 to 16 years, recruited from selected schools within the three districts. This age group is recognized as being at the highest risk of *S. mansoni* infection and associated morbidity, and is the primary target for school-based deworming programs [4, 35]. The region has an estimated 317 primary schools, providing a substantial population for sampling [37].

The study area falls under the purview of Uganda’s National Schistosomiasis Control Program (NSCP), which recommends annual preventive chemotherapy via MDA of PZQ to all school-aged children [35]. However, the implementation of MDA in this region has historically faced challenges, including logistical constraints and occasional delays in drug supply, leading to inconsistencies in coverage and timing across schools and communities [35]. This operational context likely explains the low proportion of children in our study who reported having received PZQ in the three months preceding the baseline survey, despite residing in an area targeted by the national control program.

### Study design and sampling

**Fig. 2:**
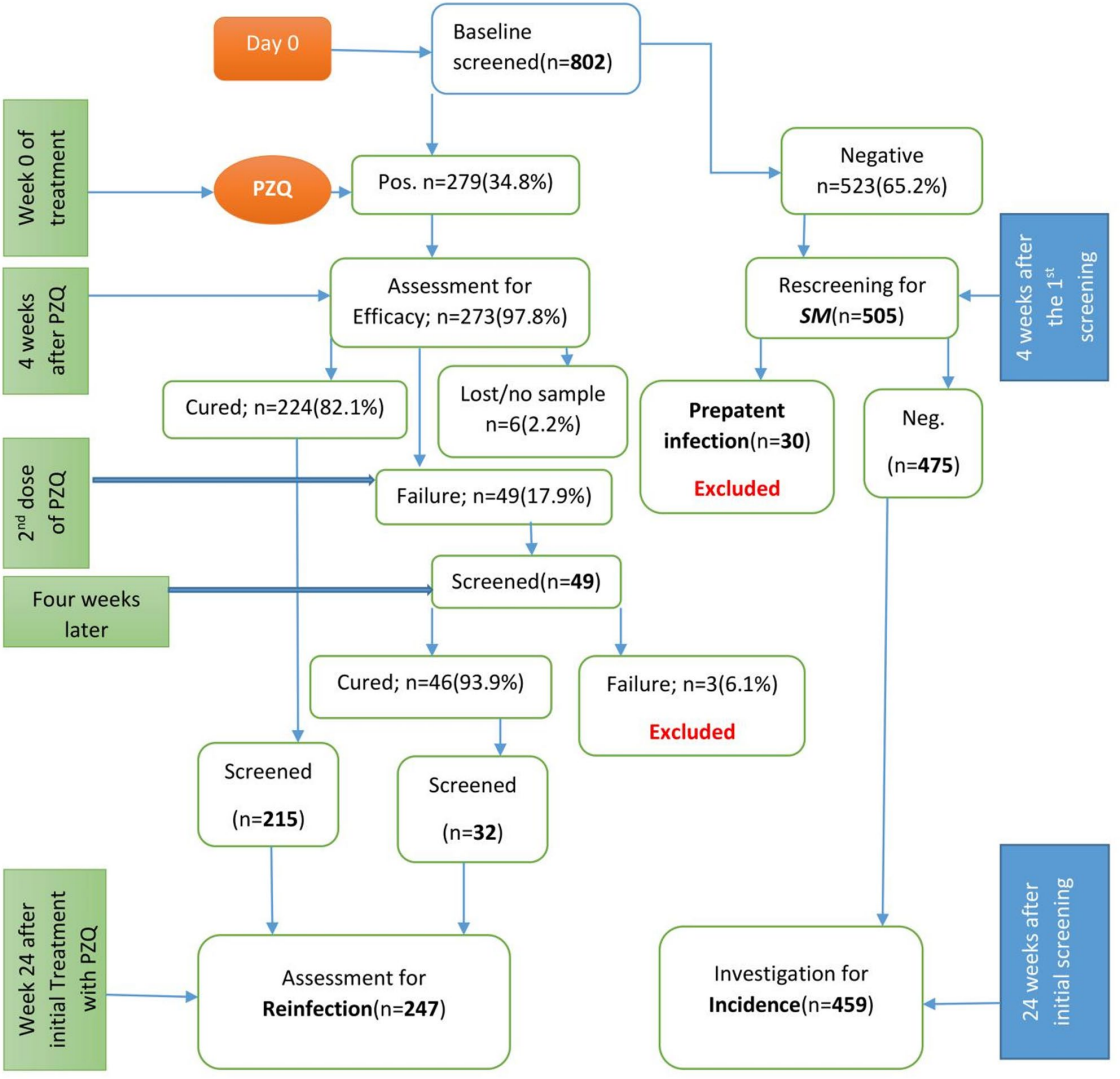
Flowchart of participant recruitment, treatment, and follow-up in a study of *Schistosoma mansoni* in primary school children in the Lango sub-region, Uganda. (*N.B: percentages are relative to the immediately preceding step*)

This study employed a prospective cohort design to investigate the efficacy of PZQ and the dynamics of reinfection and incidence of *S. mansoni* among primary school children in the Lango sub-region. The study followed two distinct cohorts over 24 weeks: an “Infected Cohort” (baseline-positive children) to assess treatment efficacy and reinfection, and an “Uninfected Cohort” (baseline-negative children) to determine the incidence of new infections (Fig. 2).

### Sample size determination

The sample size was calculated to ensure a sufficient number of infected children for the longitudinal cohort. Using the formula for cross-sectional studies by Daniel (1999) [40], which is appropriate for estimating the initial baseline prevalence required to enroll a sufficient cohort. The formula used was:

$${\bf{N}}{\rm{ }} = {\rm{ }}{{\bf{Z}}^2}{\rm{ }} \times {\rm{ }}{\bf{P}}\left({{\bf{1}} - {\bf{P}}} \right){\rm{ }}/{\rm{ }}{{\bf{d}}^2}$$

Where: Z = 1.96 (the standard normal deviate at 95% confidence level), P = anticipated prevalence (set at 50% to yield the maximum sample size), and d = 0.05 (desired precision). This calculation yielded a minimum sample of 384 to estimate a prevalence of 50%. To account for the multistage sampling design, potential non-response, and to ensure an adequately sized infected cohort, the sample was doubled and adjusting for a 10% non-response rate, resulting in a final target of 802 participants.

### Sampling technique and participant recruitment

A multistage sampling technique was used to select participants. First, a sampling frame of 317 primary schools from Lira district, Lira city, and Kole district was compiled [37]. Schools were stratified by administrative units, and the number of schools to be included from each administrative unit was allocated proportionally to the total number of schools in the administrative units. Within each administrative unit stratum, individual schools were then selected using computer-generated random numbers, yielding a total of 20 schools.

Parents or guardians of selected children provided written informed consent, and the children themselves gave assent. Participants were enrolled in the study upon providing a complete baseline stool sample.

### Study timeline and cohort formation

Following baseline screening, enrolled children were allocated into an infected cohort, which was treated and followed to assess PZQ efficacy and reinfection, and an uninfected cohort, which was followed to determine the incidence of new infections. The infected cohort was re-screened four weeks’ post-treatment to determine cure and egg reduction rates, with treatment failures receiving a second dose [41]. A final follow-up assessment for reinfection and incidence was conducted twenty-four weeks after the baseline survey (Fig. 2). Attrition was anticipated throughout the study due to absenteeism on sample collection days, failure to provide stool samples, withdrawal of consent, or transfer to another school. These attrition rates are detailed in the results section for each follow-up point.

### Stool examination and treatment

#### Sample collection and processing

All children present in the selected primary schools on the days of the survey who provided assent and had parental consent were eligible to participate. Each enrolled participant received a pre-labelled, sterile stool container marked with a unique identification code, date, and laboratory number. Participants were instructed to collect approximately 10 g of early morning stool (between 8:00 and 10:00 am) and deliver it to the on-site field laboratory within one hour of collection to ensure sample integrity.

### Parasitological examination

Stool specimens were processed using a modified Kato-Katz technique, as previously described by Odongo-Aginya et al. [36]. This method is a standard, widely used diagnostic tool for intestinal schistosomiasis in Uganda and similar settings [42, 55]. Briefly, approximately 41.7 mg of sieved stool was transferred through a standardized template onto a microscope slide. A glycerol-soaked cellophane cover slip (22 × 44 mm) was placed over the sample, and the smear was pressed to achieve a uniform thickness. To enhance visualization and differentiation of helminth eggs, a compound stain consisting of 5% eosin yellow and 7.5% nigrosin in 10% formalin was applied during preparation [36, 64]. This method has also been used in published studies from northern Uganda (34,49).

Duplicate slides prepared from the same stool sample were examined microscopically by two qualified laboratory technicians for the presence and quantification of *S. mansoni* eggs. The average number of eggs from the two duplicate slides was multiplied by 24 (since each slide contains approximately 41.7 mg of stool) to obtain eggs per gram (EPG). Infection intensity was classified according to World Health Organization guidelines as light (1–99 EPG), moderate (100–399 EPG), or heavy (≥ 400 EPG) [4]. For quality control and assurance, a senior laboratory technologist re-examined a random subset of 10% of all slides, as well as all discordant results from the first two readers, to resolve discrepancies and ensure result reliability.

### Treatment and ethical considerations

All participants who tested positive for *S. mansoni* at any point during the study were weighed using a calibrated weighing scale to determine their body weight. Based on the measured weight, each child received a single, standard oral dose of PZQ at 40 mg/kg body weight, administered as crushed tablets (600 mg each) mixed with water or porridge, free of charge. This was administered under medical supervision. Children were observed for 30 min after administration. If vomiting occurred within 1 h, the dose was repeated once. Mild adverse events (transient abdominal pain, headache) were recorded but did not require specific treatment. Children who remained positive at the 4-week follow-up (treatment failures) received a second dose of PZQ. All treatment procedures were conducted in strict accordance with the Uganda National Guidelines for the Prevention and Control of Schistosomiasis and Soil-Transmitted Helminthiasis, which are based on WHO recommendations [4, 43].

### Data management and statistical analysis

Data were double-entered, validated, and analyzed using IBM SPSS Statistics for Windows, Version 25.0 [44]. Descriptive statistics were used to summarize participant characteristics, presented as frequencies and percentages for categorical variables.

### Infection definitions and treatment efficacy

A participant was defined as positive for *S. mansoni* if at least one *S. mansoni* egg was identified on either of the two duplicate slides examined. This means that a total of appropriately 83.4 mg of stool was examined per participant (41.7 mg per slide x 2 slides). Prevalence was calculated as the number of positive individuals divided by the total number screened, multiplied by 100. Infection intensity was classified based on eggs per gram (EPG) of stool as light (1–99 EPG), moderate (100–399 EPG), or heavy (≥ 400 EPG) [4].

Praziquantel efficacy was assessed in the cohort of children positive at baseline who were available for follow-up at 4 weeks. The cure rate (CR) was defined as the percentage of treated children who became egg-negative at the 4-week follow-up [45].

$$\eqalign{ & CR = {\eqalign{ & Number\,of\,negative\,children\,after \cr & treatment\,who\,were\,positive\,at\,baseline \cr} \over \eqalign{ & Number\,of\,positive \cr & children\,before\,treatment \cr} } \cr & \times 100 \cr} $$

The **egg reduction rate (ERR)** was calculated for the group as a whole using the formula:

$$\eqalign{ & ERR \cr & = (1 - {{Arithmetic\>mean\>egg\>counts\>at\>follow\>up} \over {Arithmetic\>mean\>egg\>counts\>at\>baseline}}) \cr & \times 100 \cr} $$

### Statistical comparisons

The Pearson chi-square (χ²) test was used to assess associations between categorical variables, such as the differences in prevalence and infection intensity distribution across districts, gender, and age groups. The Wilcoxon signed-rank test was employed to compare the geometric mean egg counts before and after treatment, as the egg count data were not normally distributed [41]. A p-value of less than 0.05 was considered statistically significant for all tests.

95% confidence intervals for proportions (prevalence, cure, reinfection, and incidence rates) were calculated primarily using the Wilson score interval [46]. For subgroups with very small sample sizes (e.g., *n* ≤ 5), the Clopper-Pearson exact method was used, consistent with approaches for sparse data in contemporary epidemiological studies [47].

### Analysis of reinfection and incidence

For reinfection analysis, the cohort consisted of children who were initially positive, successfully cured after PZQ treatment (either after the first or second dose), and reassessed at 24 weeks. Reinfection was defined as a new positive test in a previously cured child at this 24-week follow-up. The reinfection rate was calculated as the number of reinfected children divided by the total number of cured children reassessed [41].

For incidence analysis, children who were negative at the initial baseline survey were re-screened four weeks later to identify prepatent infections (positive tests at 4 weeks). These individuals were treated and excluded from the incidence cohort [41]. The remaining consistently negative children were reassessed at 24 weeks. Incidence was defined as a new positive test at 24 weeks in this cohort. The incidence rate was calculated as the number of new infections divided by the number of at-risk children reassessed [48]. Pearson chi-square tests were also used to examine associations between reinfection/incidence and demographic factors. All analyses were performed on available data, with attrition rates reported for each follow-up point [41].

## Results

### *Schistosoma mansoni* prevalence and infection intensity among school children

The baseline assessment included 802 primary school children (age 5–16 years; mean ± SD: 12.26 ± 2.11 years) from the Lango sub-region, as previously described in our recent publication from the same study setting [49]. The overall prevalence of *S. mansoni* infection was 34.8% (279/802, 95% CI: 31.5–38.3).

Prevalence differed significantly across districts (χ² = 27.78, *p* < 0.001). Lira District had the highest prevalence at 44.3% (160/361), followed by Kole District at 27.9% (111/398), and Lira City at 18.6% (8/43). No significant difference in prevalence was observed by gender (Male: 36.3% [160/441] vs. Female: 33.0% [119/361]; χ² = 0.96, *p* = 0.326). However, a significant association was found with age group (χ² = 7.25, *p* = 0.027). The highest prevalence was found in children aged 9–12 years (39.8%, 144/362), compared to 30.4% (14/46) in the 5–8 years group and 30.7% (121/394) in the 13–16 years group (Table 1).

Regarding infection intensity among the 279 positive children, light infections (1–99 EPG) were the most common (45.9%, 128/279), followed by moderate (100–399 EPG; 35.8%, 100/279) and heavy (≥ 400 EPG; 18.3%, 51/279) infections. The distribution of infection intensity varied significantly by district (χ² = 82.77, *p* = 0.012). In Lira District, half of the infections were light (50.6%), whereas Kole District had a higher proportion of moderate infections (43.2%), and Lira City had a notable proportion of heavy infections (25.0%) despite the low number of cases. In contrast, infection intensity did not differ significantly by gender (χ² = 19.10, *p* = 0.895) or by age group (χ² = 61.66, *p* = 0.281).

**Table 1:** *Schistosoma mansoni* prevalence and infection intensity at baseline among primary school children in Lango sub-region, Northern Uganda

Variables	Negative	Positive	Total	X^2^	*p*-value	Infection intensity			X^2^	*p*-value
	*n*(%)	*n*(%)	*n*(%)			Light(1–99 epg)	Moderate(100–399 epg)	Heavy(≥ 400 epg)		
**Total**	523(65.2)	279(34.8)	802(100)			128(45.9)	100(35.8)	51(18.3)		
**District**				27.776	< 0.001				82.774	0.012
Lira District	201(55.7)	160(44.3)	361(45.0)			81(50.6)	52(32.5)	27(16.9)		
Kole District	287(72.1)	111(27.9)	398(49.0)			41(36.9)	48(43.2)	22(19.8)		
Lira City	35(81.4)	08(18.6)	43(5.4)			6(75.0)	0(0)	2(25)		
**Gender**				0.963	0.326				19.102	0.895
Male	281(63.7)	160(36.3)	441(55.0)			72(45.0)	60(37.5)	28(17.5)		
Female	242(67.0)	119(33.0)	361(45.0)			56(47.1)	40(33.6)	23(19.3)		
**Age groups**				7.246	0.027				61.658	0.281
5–8 years	32(69.6)	14(30.4)	46(5.7)			11(78.6)	3(21.4)	0(0)		
9–12 years	218(60.2)	144(39.8)	362(45.1)			59(41.0)	53(36.8)	32(22.2)		
13–16 years	273(69.3)	121(30.7)	394(49.1)			58(47.9)	44(36.4)	19(15.7)		

Values are n (%) unless otherwise indicated. Overall prevalence: 34.8% (95% CI:31.5–38.3)

### Praziquantel efficacy against *Schistosoma mansoni*

The flowchart of participant recruitment, treatment, and follow-up is shown in Fig. 2. The efficacy of a single 40 mg/kg dose of PZQ was assessed four weeks post-treatment in 273 of the 279 children who were positive at baseline (attrition rate: 2.15%). The overall cure rate (CR) after the first dose was 82.1% (224/273, 95% CI: 77.0-86.4), with 49 children (17.9%) classified as treatment failures.

Cure rates were consistently high across all demographic subgroups after the first dose (Table 2). By district, CRs were 81.8% (126/154) in Lira District, 81.1% (90/111) in Kole District, and 100% (8/8) in Lira City. There was little difference in CR by gender, with males at 80.1% (125/156) and females at 84.6% (99/117). By age, the highest CR was observed in the 5–8 years’ group (92.9%, 13/14), compared to 81.7% (116/142) in 9–12 years and 81.2% (95/117) in 13–16 years.

When stratified by baseline infection intensity, cure rates were highest among children with light infection (91.4%, 117/128), followed by moderate (78.0%, 78/100), and heavy (66.7%, 34/51) infections (Additional File 1). This pattern was consistent across districts.

Despite the high cure rates, the egg reduction rates (ERR) varied considerably between subgroups (Table 2). The overall ERR after the first dose was 32.9%. Modest reductions were observed in Lira District (ERR: 15.8%), in the 13–16 years’ age group (ERR: 19.4%), and among males (ERR: 25.4%), while more substantial reductions were seen in Kole District (ERR: 47.4%) and the 5–8 years’ age group (ERR: 73.1%). A Wilcoxon signed-rank test confirmed a highly significant reduction in geometric mean egg counts from baseline (geometric mean: 89.2 EPG) to after the first dose (geometric mean: 24.6 EPG) (Z = -6.103, *p* < 0.001).

The forty-nine (49) first-dose failure cases received a second PZQ dose, after which 93.9% (46/49, 95% CI: 83.6–97.9) were cured. This second dose raised the CR to 95.2% (20/21) in Kole District and 92.9% (26/28) in Lira District. Similarly, CRs increased to 93.5% (29/31) in males and 94.4% (17/18) in females. The three children (6.1%) who remained positive after the second dose were considered final treatment failures and were referred to a health facility for further management (Table 2). The egg reduction after the second dose was substantial, with the overall AM intensity among re-treated children falling to low levels (e.g., 36 EPG in Lira District, 48 EPG in Kole District). However, the reduction in geometric mean egg counts from baseline (geometric mean: 156.3 EPG) to after the second dose (geometric mean: 28.4 EPG) was not statistically significant (Z = -1.604, *p* = 0.109).

In summary, a single dose of PZQ was highly effective at achieving parasitological cure (~ 80–100%) across all subgroups. A second dose administered to initial non-responders further increased the overall cure rate, successfully resolving the majority of persistent infections.

**Table 2:** Praziquantel (PZQ) efficacy against *Schistosoma mansoni* after the first and second dose at 40 mg/kg

Variables		Baseline		4 weeks after 1st dose of PZQ						4 weeks after 2nd dose of PZQ					
	No. tested	Positive	AM of EPG	No. tested	No. cured	Failure case	CR	AM of EPG	ERR	No. tested	No. cured	Failure cases	CR	AM of EPG	ERR
**District**															
Lira District	361	160	191.4	154	126	28	81.8	161.14	15.8	28	26	02	92.9	36	77.7
Kole District	398	111	217.3	111	90	21	81.1	114.3	47.4	21	20	1	95.2	48	58.0
Lira City	43	8	300	08	08	00	100	00	100	0	NC	NC	NC	NC	NC
**Gender**															
Male	441	160	198.3	156	125	31	80.1	147.9	25.4	31	29	02	93.5	36	75.7
Female	361	119	204.7	117	99	18	84.6	129.3	36.8	18	17	1	94.4	48	62.9
**Age groups**															
5–8 years	46	14	89.1	14	13	1	92.9	24	73.1	1	1	0	100	0	100
9–12 years	362	144	222.9	142	116	26	81.7	136.6	38.7	26	24	2	92.3	48	64.9
13–16 years	394	121	188.0	117	95	22	81.2	151.6	19.4	22	21	1	95.5	24	84.2
Overall	802	279	204.9	273	224	49	82.1	137.4	32.9	49	46	3	93.9	41.1	NC

Abbreviations: AM, Arithmetic Mean (EPG); CR, Cure Rate (%); ERR, Egg Reduction Rate (%); NC, Not Calculable. Note: For Lira City second dose, no children were tested (*n* = 0), so all subsequent columns are marked NC

### Reinfection, prepatent infections, and incidence

The longitudinal dynamics of *S. mansoni* infection were assessed over 24 weeks following treatment.

### Reinfection

Reinfection was evaluated in the cohort of children who were successfully cured after treatment (either after the first or second PZQ dose) and available for follow-up at 24 weeks. Of the 224 children cured after the first dose, 215 were reassessed (attrition: 4.0%), of whom 16 (7.4%) had become reinfected. Of the 46 children cured after the second dose, 32 were reassessed (attrition: 30.4%), of whom 3 (9.4%) were reinfected. Combining these groups, the overall reinfection cohort consisted of 247 children. The overall reinfection rate was 7.7% (19/247, 95% CI: 4.9–11.8), meaning 92.3% (228/247) of initially positive children remained cured six months post-treatment. As shown in Table 3, reinfection rates varied modestly by district, gender, and age group. However, Pearson chi-square tests confirmed that none of these differences were statistically significant (District: χ² = 0.363, *p* = 0.834; Gender: χ² = 0.313, *p* = 0.576; Age Group: χ² = 1.136, *p* = 0.567).

### Prepatent infections and incidence of new infections

To accurately measure new infections, we first identified and treated prepatent infections (those not detectable at baseline but patent four weeks later). Out of 523 children negative at baseline, 505 were re-screened (attrition: 3.4%). Of these, 5.9% (30/505, 95% CI: 4.1–8.3) were positive, indicating prepatent infections at baseline. These children were treated and excluded from the incidence analysis (Table 3).

The true incidence of new infections was then assessed in the remaining cohort of 475 consistently negative children. At the 24-weeks follow-up, 459 children were screened (attrition: 3.4%). The overall incidence of new *S. mansoni* infections was 5.0% (23/459, 95% CI: 3.3–7.4), with 95.0% (436/459) remaining negative. The incidence of infection showed a significant association with district (χ² = 32.48, *p* < 0.001), with a notably high rate observed in Lira City (100%, 2/2). However, this estimate is based on an extremely small sample size (only 2 children at risk), and the 95% confidence interval is very wide (34.2–100.0%). Therefore, this finding should be interpreted with extreme caution and does not necessarily indicate that Lira City has a higher true incidence than other districts. No significant associations were found with gender (χ² = 0.140, *p* = 0.708) or age group (χ² = 1.905, *p* = 0.386).

**Table 3:** Reinfection, prepatent, and incidence of *Schistosoma mansoni* 24 weeks post-baseline, with 95% confidence intervals and p-values

Variable	Category	Reinfection Cohort *(Initially positive and cured)*	*p*-value	Prepatent Infection (Initially negative, 4-week follow-up)	*p*-value	Incidence Cohort (Consistently negative, 24-week follow-up)	*p*-value
		No. at Risk	Reinfected *n* (%, 95% CI)	No. Screened	Prepatent *n* (%, 95% CI)	No. at Risk	New Infection *n* (%, 95% CI)
**Overall**		247	19 (7.7, 4.9–11.8)	505	30 (5.9%, 4.1–8.3)	459	23 (5.0%, 3.3–7.4)
**District**	Lira District	141	10 (7.1%, 3.6–12.7)	184	17 (9.2%, 5.6–14.4)	186	10 (5.4%, 2.7–9.7)
	Kole District	98	8 (8.2%, 3.8–15.5)	264	11 (4.2%, 2.2–7.3)	271	11 (4.1%, 2.1–7.1)
	Lira City	8	1 (12.5%, 0.6–47.1)	27	2 (7.4%, 1.3–23.3)	2	2 (100.0%, 34.2–100.0)
			*p* = 0.834		*p* = 0.056		*p* < 0.001
**Gender**	Male	145	10 (6.9%, 3.5–12.3)	255	16 (6.3%, 3.7–10.1)	242	13 (5.4%, 3.0-8.9)
	Female	102	9 (8.8%, 4.3–16.2)	220	14 (6.4%, 3.6–10.5)	217	10 (4.6%, 2.3–8.3)
			*p* = 0.576		*p* = 0.708		*p* = 0.708
**Age Group**	5–8 years	12	0 (0.0%, 0.0-24.9)	29	2 (6.9%, 1.2–21.9)	31	2 (6.5%, 1.1–20.7)
	9–12 years	131	10 (7.6%, 3.9–13.7)	199	12 (6.0%, 3.2–10.3)	204	13 (6.4%, 3.5–10.6)
	13–16 years	104	9 (8.7%, 4.3–15.7)	247	16 (6.5%, 3.8–10.4)	224	8 (3.6%, 1.6–6.9)
			*p* = 0.567		*p* = 0.386		*p* = 0.386

* Confidence interval for Lira city incidence calculated using Clopper-Pearson exact method due to small sample size (*n* = 2). All other 95% CIs using the Wilson score method

## Discussion

This 24-week cohort study provides a detailed assessment of the baseline epidemiology, treatment efficacy, and post-treatment dynamics of *S. mansoni* among schoolchildren in the Lango sub-region of Northern Uganda. The measurable rates of reinfection (7.7%) and incidence (5.0%) within six months post-treatment, together with the high baseline prevalence (34.8%), underscore persistent high transmission intensity in this focus. Understanding true incidence after excluding prepatent infections is critical because a single baseline survey can miss incubating infections, leading to overestimation of incidence [4, 31, 32]. By re-screening at 4 weeks and treating prepatent cases, our study provides a more accurate estimate of new infections.

The overall baseline prevalence of *S. mansoni* was 34.8%(95% CI: 31.5–38.3), confirming its status as a major public health problem in the Lango sub-region, consistent with findings from other highly endemic areas in Uganda and post-conflict reassessments [33, 49, 50, 54]. Our observed rate is higher than the 21.6% reported by Tinkitina et al. in preschool-aged children from the same sub-region, a difference likely attributable to the older age group in our study, which has higher cumulative exposure [51]. The significant variation in prevalence by district (Lira: 44.3%, Kole: 27.9%, Lira City: 18.6%) reflects micro-epidemiological heterogeneity driven by proximity to water bodies, snail host distribution, and local water contact behaviors [52, 53]. This heterogeneity does not bias our efficacy estimates but rather strengthens external validity by showing that PZQ remains highly effective across diverse transmission settings.

The overall cure rate of 82.1% (95% CI: 77.0-86.4) falls within the range reported in recent systematic reviews and meta-analyses of PZQ efficacy in East Africa. For example, Sisay et al. (2025) reported pooled CRs ranging from 70.2% to 88.6% depending on transmission intensity [17], and Zwang & Olliaro (2014) found an average CR of 77.1% (95% CI: 68.8–85.4) for *S. mansoni* after a single 40 mg/kg dose [16]. Our CR of 82.1% confirms that PZQ remains highly effective in the Lango sub-region despite over a decade of MDA implementation. The primary goal of treatment is to prevent morbidity, and studies in Uganda have shown that PZQ treatment can lead to sustained reductions in hepatosplenic pathology and associated morbidity over the long term, underscoring its clinical value beyond immediate parasitological clearance [57–59]. The superior CR in the 5–8 years-age group (92.9%) may be due to lower initial worm burdens or differing immune responses, as also observed by Crellen et al. [24]. The observation that cure rates decreased with increasing baseline infection intensity (light: 91.4%, moderate: 78.0%, heavy: 66.7%) aligns with the previous reports and underscores the importance of repeated treatment rounds to clear heavy infections (Additional File 1).

Despite high CRs, the variable and sometimes modest egg reduction rates (ERR) across subgroups (ranging from 15.8% to 73.1%) warrant discussion. The low ERR in Lira District (15.8%) and among males (25.4%) could reflect high baseline intensities where arithmetic means are heavily influenced by outliers with very high egg counts who remain infected. Alternatively, it might indicate a residual worm population that continues to produce eggs despite reduced worm burden. Repeated observation of low ERR over multiple treatment rounds could signal emerging PZQ tolerance, as suggested by Crellen et al. (2016) and Eastham et al. (2024) [23, 24]. However, our high CR argues against frank resistance. We recommend that future studies monitor both CR and ERR over time to detect any trends suggesting declining drug efficacy.

The success of a second PZQ dose, which cured 93.9% of initial treatment failures, provides compelling evidence for its use in test-and-re-treat strategies. This finding is crucial for program managers; a second dose could be a highly effective tool for managing persistent infections in high-risk individuals, improving overall control outcomes and potentially slowing the development of drug tolerance [4, 26]. From a programmatic perspective, adding a second dose increases drug costs and requires follow-up visits. However, the benefits of reducing the infectious reservoir, treating high-burden children who contribute disproportionately to environmental contamination, and preventing chronic morbidity may outweigh these costs in high-transmission settings [24]. Future implementation research should evaluate the cost-effectiveness of school-based test-and-re-treat protocols compared to standard annual MDA.

The 24-week reinfection rate (7.7%) and incidence rate (5.0%) provide a clear measure of ongoing transmission pressure. The significant association between incidence and district (*p* < 0.001) points to localized hotspots, but the very high incidence estimate for Lira City (100%) is based on only two children and must be interpreted with extreme caution, as it does not necessarily indicate higher true incidence in that district. Our reinfection rate is lower than the 19.2% reported over six months in a high-transmission focus in Senegal [60], likely due to differences in snail ecology, water-contact behaviors, and environmental control measures. The lack of significant association between reinfection and demographic factors suggests that exposure is widespread and relatively uniform across groups in these endemic communities.

Identification of prepatent infections in 5.9% of children initially negative children is a critical methodological strength of this study. By treating and excluding these individuals, we avoided overestimating the true incidence, a common pitfall in longitudinal schistosomiasis studies that has been highlighted by researchers like Kittur and others [61–63]. This finding emphasizes that a single baseline survey can miss a substantial proportion of incubating infections.

### Limitations of the study

The interpretations of this study are subject to several limitations. First, we observed notable attrition rate in the group that required a second PZQ dose (30.4%), which could have introduced selection bias. We did not apply inverse probability weighting or other statistical adjustments for attrition, as the primary analysis was descriptive and attrition rates were low in most follow-up points (2.2-4.0%). However, the higher attrition in the second-dose subgroup may limit the generalizability of findings for this specific population. Future studies should implement strategies to minimize attrition and consider sensitivity analyses (e.g., inverse probability weighting) to assess the impact of missing data.

Second, we lacked detailed data on water contact patterns and snail intermediate host densities, which would have helped explain the observed micro-epidemiological heterogeneity in reinfection and incidence rates. Collecting such data in future studies using structured questionnaires and environmental mapping would enable a better understanding of transmission risk factors and could guide targeted interventions (Reitzug et al., 2024; Trienekens et al., 2022 [25, 27]).

Third, the Kato-Katz technique has well-documented limitations in sensitivity, particularly for low-intensity infections. Studies have shown that a single Kato-Katz smear (41.7 mg) has a sensitivity of approximately 50–70% for detecting *S. mansoni* infections compared to multiple-sample reference standards, with sensitivity dropping to below 30% for infections with < 50 EPG [56]. This means that some light infections (1–99 EPG) may have been missed at baseline, potentially leading to underestimation of true prevalence and overestimation of incidence. The use of duplicate slides from the same sample improves sensitivity only modestly (**≈** 10–15%) compared to examining two separate stool samples collected on different days [56]. We acknowledge this limitation and its potential impact on our reinfection and incidence estimates.

Fourth, we were unable to provide confidence intervals for the overall egg reduction rate (ERR) due to the presentation of efficacy data as group-level arithmetic means without corresponding individual-level variance measures. Future studies should report individual egg counts to enable calculation of ERR with appropriate confidence intervals.

Fifth, we collected only a single stool sample per participant rather than multiple consecutive samples. While this approach is common in large-scale field studies in resource-limited settings, collecting three consecutive samples would have improved diagnostic sensitivity, particularly for detecting low-intensity infections [56]. However, we mitigated this limitation by examining duplicate slides from each sample and using the modified nigrosin-eosin staining technique, which enhances egg visualization [36].

Sixth, the variable egg reduction rates observed across districts and demographic subgroups (ranging from 15.8% to 73.1%) raise the question of whether these differences reflect true variation in drug efficacy, differences in baseline infection intensity, or potential early signals of emerging PZQ tolerance. While our cure rates remained high (82.1% after the first dose), low ERR in some subgroups could occur if a small number of individuals with very high baseline egg counts remain infected and continue to excrete large numbers of eggs. However, repeated observation of low ERR in the same population over multiple treatment rounds could indicate the reduced PZQ susceptibility [24, 23]. We did not have individual-level longitudinal data across multiple treatment rounds to assess this possibility, but we recommend that future studies monitor both CR and ERR over time to detect trends suggesting declining drug efficacy.

Seventh, the findings from Lira City, particularly the 100% incidence rate observed in the very small cohort of two children, should be interpreted with extreme caution. This estimate is statistically unstable (95% CI: 34.2–100.0%) and may not reflect the true incidence in this district. The small sample from Lira City (only 5.4% of the total study population) limits our ability to draw meaningful conclusions about transmission dynamics in this specific area.

### Implications for control and elimination

The measurable rates of reinfection and incidence six months post-treatment indicate that annual MDA, while reducing prevalence and morbidity, may be insufficient to interrupt transmission in this setting. To advance from morbidity control towards transmission interruption, we recommend piloting more frequent drug administration (e.g., biannual MDA) and integrating targeted test-and-re-treat approaches within school-based programs. The high efficacy of a second PZQ dose supports its use for non-responders, but program managers must consider the cost-effectiveness trade-off: test-and-re-treat requires diagnostic testing (adding cost and logistics), whereas empirical retreatment of all positives avoids testing costs but may waste drugs and accelerate tolerance.

In high-transmission settings where reinfection is rapid, empirical retreatment of non-responders (e.g., treating all children who remain positive after the first dose without re-testing) may be more operationally feasible, while test-and-re-treat may be preferable where rapid diagnostic tests (e.g., POC-CCA) become affordable [4]. Evidence from other East African programs (e.g., Zanzibar, Kenya) shows that biannual MDA can reduce prevalence more effectively than annual schedules [14, 15]. Complementing chemotherapy with Water, Sanitation, and Hygiene (WASH) interventions and snail control is essential for sustained impact [4, 29, 51].

## Conclusion

In conclusion, this 24-week cohort study demonstrates that a single dose of PZQ provides high parasitological cure against *S. mansoni* in northern Uganda, and a second dose effectively rescues most initial treatment failures. However, the substantial infection burden at baseline, combined with measurable reinfection (7.7%) and incidence (5.0%) rates within six months, underscores persistent high transmission intensity. To advance from morbidity control towards interruption of transmission in this focus, a revision of the current annual MDA strategy is warranted. We recommend piloting more frequent drug administration, integrating targeted test-and-re-treat approaches (alongside careful cost-effectiveness evaluation), and robustly implementing complementary WASH interventions and snail control.

## Supplementary Information

Below is the link to the electronic supplementary material.

Supplementary Material 1: Additional File 1: Cure rates after first dose of praziquantel (40 mg/kg) stratified by baseline infection intensity among school-aged children in Lango sub-region, Uganda

## Data Availability

The datasets used and/or analyzed during the current study are available from the corresponding author upon reasonable request.

## Abbreviations

AM: Arithmetic Mean
CR: Cure Rate
DALYs: Disability-Adjusted Life Years
EPG: Eggs per gram
ERR: Egg Reduction Rate
MDA: Mass Drug Administration
NTDs: Neglected Tropical Diseases
NSCP: National Schistosomiasis Control Program (Uganda)
PZQ: Praziquantel
SD: Standard Deviation
SPSS: Statistical Package for the Social Sciences
WASH: Water, Sanitation, and Hygiene
WHO: World Health Organization
