# Supplementary material for: Praziquantel efficacy and post-treatment reinfection and incidence of Schistosoma mansoni among school-aged children in Northern Uganda: a 24-week cohort study in a high transmission focus
Source: BMC Infect Dis. 2026 Jun 6;26:1475. doi: 10.1186/s12879-026-13757-x (PMC13471520; doi:10.1186/s12879-026-13757-x)
Supplement: Supplementary file 1 [file 12879_2026_13757_MOESM1_ESM.docx]

**Additional File 1:** Cure rates after first dose of praziquantel (40 mg/kg) stratified by baseline infection intensity among school-aged children in Lango sub-region, Uganda

| **District** | **Infection intensity** | **Number treated** | **Number cured** | **Cure rate (%)** | **95% CI** |
| --- | --- | --- | --- | --- | --- |
| Lira District | Light (1-99 EPG) | 79 | 74 | 93.7 | 85.8-97.5 |
|  | Moderate (100-399 EPG) | 52 | 40 | 76.9 | 63.2-86.6 |
|  | Heavy (≥400 EPG) | 27 | 16 | 59.3 | 39.9-76.4 |
| Kole District | Light | 40 | 38 | 95.0 | 82.7-99.2 |
|  | Moderate | 48 | 38 | 79.2 | 65.0-88.9 |
|  | Heavy | 22 | 14 | 63.6 | 41.7-81.2 |
| Lira City | Light | 6 | 6 | 100 | 56.6-100 |
|  | Moderate | 0 | - | - | - |
|  | Heavy | 2 | 2 | 100 | 34.2-100 |
| **Overall** | **Light** | **128** | **117** | **91.4** | **85.1-95.3** |
|  | **Moderate** | **100** | **78** | **78.0** | **68.7-85.2** |
|  | **Heavy** | **51** | **34** | **66.7** | **52.5-78.4** |

**Note:** *Cure rate decreased significantly with increasing infection intensity (χ² trend = 18.9, p < 0.001).*
